# Alternative treatment modality for severe aplastic anemia in a resource-limited setting: a single-institution prospective cohort study from Upper Egypt

**DOI:** 10.1007/s00277-023-05440-x

**Published:** 2023-09-13

**Authors:** Mervat A M Youssef, Mohammed H Ghazaly, Mai A Abdelfattah

**Affiliations:** Children Hospital, Hematology Unit, Faculty of Medicine, Assiut University, Assiut, Egypt

**Keywords:** Severe aplastic anemia, Children, Cyclosporin, Eltrombopag

## Abstract

This study compared the efficacy and safety of CsA monotherapy with eltrombopag (E-PAG) + CsA combined treatment in children with severe aplastic anemia (SAA). The study including 30 children had SAA. Ten were a retrospective cohort treated with CsA monotherapy. The other 20 were prospective cohort received E-PAG + CsA. All patients were evaluated for partial (PR) and complete (CR) hematological response at 3, 6, and 12 months. overall response (OR), overall survival rates (OS), and treatment safety. OR for the E-PAG patients was 40% after 3 months of therapy. At 6 months, this had increased to 75% with significantly higher CR rate (40%) than in the CsA group (*p* = 0.0001). After a year of treatment, the CR for the E-PAG + CsA regimen had increased to 50% and the OR to 85%, compared to 20% in the CsA group (*p* = 0.0001). The OS at 12 months was 100% in the E-PAG+ CsA group compared to 80% in the CsA cohort. At 24 months, the OS in the E-PAG + CsA group was 90%. In conclusion, E-PAG+ CsA was found to be a safe and effective alternative treatment for children with SAA particularly in countries with limited resources.

## Introduction

Aplastic anemia (AA) is a life-threatening condition characterized by pancytopenia and hypocellular bone marrow but without major dysplastic symptoms or marrow fibrosis [1, 2]. The incidence of acquired AA is about two children per million each year in Europe and North America but this number is 2–3 times higher in East Asia [2, 3]. AA affects both genders equally and can occur at any age. However, it is slightly more common during childhood, and 50% of cases occur in the first three decades of life [4, 5]. The pathogenesis of AA is multifactorial and may involve an abnormal hematopoietic microenvironment, hematopoietic stem cell/progenitor cell deficiencies, immunity disorders, or mutation of the genes responsible for hematopoiesis. Any of these factors can cause damage or primary defects of the stem cells or marrow microenvironment [6].

It can be difficult to differentiate between the acquired and inherited forms of this disease. Inherited causes are responsible for about 25–30% of pediatric cases of AA5. Acquired aplastic anemia may be idiopathic (>80%), post-infection (15% [particularly after hepatitis, Epstein-Barr virus, human immune deficiency virus, parvovirus, and mycobacteria]), or toxin/ drug-induced (4%) [7, 8].

Hematopoietic stem cell transplantation (HSCT) from a human leukocyte antigen (HLA)-matched sibling donor is the definitive curative therapy for AA [9, 10]. The major drawback of HSCT is that only 30% of patients have a suitably matched donor. Also, there is a risk of graft-versus-host disease (GVHD), which can cause mortality or morbidities with long-term effects on quality of life [11]. Unfortunately, allogeneic transplantation is not possible in most developing countries. The alternative treatment for AA in about two-thirds of cases is immunosuppressive therapy (IST) consisting of anti-thymocyte globulin (ATG) (horse or rabbit) and cyclosporine (CsA) [12, 13]. IST carries a satisfactory long-term response but 30–40% of patients do not respond and pancytopenia [9 ]or thrombocytopenia can continue after therapy, even in cases with improvements in life-threatening neutropenia [14]. HSCT and IST regimens can control the manifestation of AA effectively but both have limitations. HSCT is very expensive and requires a suitable donor. Many patients do not meet the requirements for HSCT. Yet, IST can leave a significant number of patients with persistent cytopenia.

CsA is sometimes used alone as a monotherapy in countries with poor resources [15, 16]. It is an effective immunosuppressant, inexpensive, accessible, can be administered to outpatients and is less toxic than combined treatment with ATG. The response rate to CsA monotherapy is between 30% and 50% [17, 18]. Thrombopoietin (TPO) is a glycoprotein class 1 hematopoietic cytokine, primarily manufactured in the liver [19]. It is an important regulator of hematopoiesis [20]. It acts through c-Mpl TPO receptors expressed in hematopoietic stem cells and progenitor cells. TPO causes signal transduction events that prevent apoptosis, improve cell viability, promote growth and possibly increase differentiation [21]. Eltrombopag (E-PAG) is an oral thrombopoietin mimetic that selectively binds to c-Mpl at the transmembranes and juxtamembranes of TPO receptors. It can circumvent the inhibitory effect of interferon-γ on HSCs and signal the c-MPL to yield a stimulatory effect. Noteworthy, interferon-γ found to have an inhibitory effect on the endogenous TPO by forming a heterodimer-hindering signalling through c-MPL and consequently

E-PAG promotes thrombopoiesis, the release of platelets from mature megakaryocytes [22], and other forms of hematopoietic stem cell differentiation [19–21, 23, 24]. E-PAG was approved by the Drug Administration (FDA) in 2008 as the first oral platelet growth factor treatment for adults with chronic immune thrombocytopenic purpura (ITP). In 2015, this approval was extended to include children aged 1 to 17 with chronic ITP. It has recently demonstrated excellent results as a treatment for AA, with trilineage responses in some patients and transfusion independence in many [25, 26]. It was licensed by the European Medicines Agency for AA in 2015. In 2017, the National Institutes of Health made E-PAG a standard of care in AA [27].

To the best of our knowledge, no previous research has compared the efficacy and safety of E-PAG + CsA with that of CsA alone in pediatric patients with SAA. We designed this study to explore the effectiveness and safety of eltrombopag added to CsA in pediatric severe aplastic anemia (SAA)

## Methods

### Design

This was a prospective, single-center clinical trial conducted at Assiut University Children’s Hospital in Egypt. The study enrolled 30 SAA patients between 1 and 18 years old. Our sample comprised two groups. Ten patients (CsA group) were a historical cohort who received cyclosporine (CsA) monotherapy from August 2016 to February 2018 because ATG was unavailable for financial reasons and E-PAG had not yet been approved by health insurers when these patients were treated. This cohort was our comparison group. Twenty patients fulfilled the eligibility criteria^x^ were recruited prospectively in the study from October 20 19 to August 2021 and treated with E-PAG + CsA (E-PAG + CsA group). All patients were evaluated for their hematological response to treatment, and to determine complete response (CR), partial response (PR), and Overall response (OR) rates and treatment safety after 3, 6, and 12 months of treatment. Pre-treatment evaluations included a complete medical history and physical examination, complete blood count (CBC) with differential, serum chemistry, bone marrow aspiration and biopsy, viral serology, immunological tests, flow cytometric tests, a diepoxybutane clastogenic stress assay, and inherited bone marrow failure panel and HLA typing. Patient follow-ups were performed every 2–4 weeks and included a CBC and monitoring of kidney and liver function.

### Eligibility criteria

Children with newly diagnosed and previously untreated SAA and adequate hepatic and renal functions who met the standard guidelines for the diagnosis and treatment of pediatric AA [28} and the modified Camitta criteria for SAA were eligible for inclusion in this study [28]_._ According to these criteria, a diagnosis of SAA may be made if bone marrow cellularity is <25% and/or at least two of the following criteria are met: (i) the absolute neutrophil count is below 0.5 × 109/L, (ii) the platelet count is below 20 × 109/L, (iii) the reticulocyte count is below 20 × 109/L.

The exclusion criteria were inherited bone marrow failure, myelodysplasia, AA secondary to infection or organ failure, underproduction anaemias secondary to B12, folate or iron deficiency, or with other reversible causes. Patients with documented hypersensitivity to any of the component medications were also excluded. The study was approved by Assiut University’s Ethical Committee for Clinical Research and informed consents were obtained from the guardians of trial participants before the study.

### Treatment plan

Patients aged 1–5 years received an initial oral dose of E-PAG of 25 mg once daily. Those aged >5 years received an initial daily dose of 50 mg/day [29]. Dose was escalated by 25 mg every two weeks in all patients, and then maintained at the maximum dose when it was reached. In patients aged 1–5, the maximum dose was 75 mg; in patients over five, it was 150 mg. Adjustments and reductions of the E-PAG dose were made where necessary based on the pharmacokinetic data for ITP [30]. Patients experienced distinct adverse events in response to treatment were excluded from the study.

Oral CsA treatment in both groups was initiated at 5–10 mg/kg/day and the dose adjusted to maintain trough levels of 170–270 ng/ml. CsA was continued for at least 12 months as tolerated and, in those who responded, continued at a fixed daily dose for at least an additional 6 months before weaning. Serum CsA levels were measured every 2 to 4 weeks while patients were receiving the drug [29].

### Supportive therapy

Supportive therapy was allowed for both cohorts throughout the study when required. This included granulocyte colony-stimulating factor (G-CSF), iron chelation, or platelet transfusion (if the count was <10,000/μL with an apparent bleeding tendency or <20,000/μL with fever) and red blood cell (RBC) transfusion (if hemoglobin was <7 g/dL or in the presence of significant symptoms, such as exertional dyspnea or anemic heart failure).

### Primary outcome measures

The primary outcomes were safety, hematological response either CR or PR, and OR rates of combined E-PAG + CsA treatment after 3, 6, and 12 months, using the standard guidelines for the diagnosis and treatment of pediatric AA [31].

### Response criteria

A hematological response was defined as a platelet count increase of at least 20 000/μL and/or platelet transfusion independence for a minimum of 8 weeks, a hemoglobin level increase of at least 1.5 g/L or a reduction in the number of PRBCs units transfused by at least four for eight consecutive weeks (compared with transfusion requirements during the 8 weeks preceding study treatment onset) and an increase of absolute neutrophil count (ANC) of >500/μL in patients with a pre-treatment count <500/μl. A PR was defined as a blood count no longer meeting the Camitta criteria for SAA and no transfusion dependence for platelets or red blood cells . A CR was defined as Hb levels of ≥100 g/l, a platelet count ≥100 × 109/L, ANC of ≥1 × 109/L and transfusion and growth factor independence. OR rates included all PR and CR within each group.

### Secondary outcome measures

Secondary outcomes were the tolerability and toxicity of the E-PAG + CsA combination.

### Statistical analysis

Data analyses were carried out using SPSS version 20. Descriptive statistics were expressed as frequencies and percentages for categorical data. Continuous variables were expressed as medians and interquartile ranges (IQR Q1 to Q3) as the sample size was small. Categorical data were compared using z score tests when the expected frequencies were less than five. The Mann-Whitney *U* test was used to determine differences in continuous variables between groups. A *p*-value of <0.05 was deemed statistically significant.

## Results

### Patient characteristics

A total of 30 patients were enrolled in this study. All patients were negative for Fancony anemia by Chromosome breakage analysis (CBA) and no one had any genetic mutations on the inherited bone marrow failure panel. Also all were negative for paroxysmal nocturnal hemoglobinuria (PNH) by Flow cytometry.

Ten of these received CsA alone (CsA group) and the other 20 received E-PAG + CsA (E-PAG + CsA). All children achieved adequate serum levels of cyclosporine.

The demographic and clinical characteristics and baseline CBC of the groups are shown in Table 1. Age and sex were matched between groups.

**Table 1:** Demographic characteristics of patients in each group

Character	CsA (*n*= 10)	E-PAG + CsA group (*n*= 20)	*P*-value
Age (median)	10.5 (7.5-12.7)	11.5 (6.5-12.5)	0.97
Sex			
Male	9 (90%)	12(60%)	0.12
Female	1 (10%)	8 (40%)	
Baseline CBC			
ANC (cell/ul)	260 (196.7-636.2)	324 ((171-478)	0.94
RETIC (%)	0.25 (0.1- 0.4)	0.4 (0.1- 0.8)	0.41
Hg (g/dl)	4.25 (4-5)	5 (4.7- 5)	0.11
PLT (10^9^/L)	8.5 (5.7-19.7)	8.5 (6.7-16.7)	0.93
PNH (No)	**0**	**0**	
Marrow cellularity			
<10% (No.)	**2 (20%)**	**6 (30%)**	
10–20% (No.)	**4 (4%)**	**10 (50%)**	
20–30% (No.)	**4 (4%)**	**4 (20%)**	

*PNH*, paroxysmal nocturnal hemoglobinuria; *ANC*; absolute neutrophil count; *Hb* Hemoglobin (g/L); *PLT* platelet count; *RETIC* absolute reticulocytic count. Data are expressed as the number of cases (%) or median with inter-quartile range. Note: *p*-value is calculated to compare both cohorts using Mann-Whitney test

There was no significant difference between the groups in the baseline CBC. Bone marrow biopsies showed <10% nucleated cell proliferation in eight patients, 10–20% in fourteen patients, and 20–30% in eight patients.

### Hematological response

Summaries of the hematological responses of both groups before treatment and after 3, 6, and 12 months of treatment are provided in Tables 2 and 3. All 30 patients were dependent on platelet and RBC transfusions before the treatment regimen began.

**Table 2:** Summary of response rate of E-PAG+ CsA group at 3, 6, 12, and 24 months

N	Age (yr)	Sex	Before E-PAG			At 3 months of E-PAG			Response	At 6 month of E-PAG			Response	At 12 month of E-PAG			Response	SE	At 24 month of E- PAG			Response
			Hb (g/dl)	ANC (cell/ul)	PLT (x10^9^/L)	Hb (g/dl)	ANC (cell/ul)	PLT (x109/L)		Hb (g/dl)	ANC (cell/ul)	PLT (x10^9^/L)		Hb (g/dl)	ANC (cell/ul)	PLT (x10^9^/L)			Hb (g/dl)	ANC (cell/ul)	PLT (x109/L)	
1	12	F	5	759	20	8	1440	125	PR	10	1450	138	CR	11	1800	180	CR	RB	11	2200	165	CR
2	14	M	3	465	7	9	1650	80	PR	11	1800	110	CR	12	2000	110	CR	He,RB	13.3	3600	100	CR
3	11	F	5	200	8	7	800	30	NR	7.8	810	90	PR	11	2300	150	CR		12	4266	220	CR
4	7	F	6	330	19	7.5	590	40	NR	8	600	80	PR	8	700	100	PR	H ,RB	10.9	1124	76	PR
5	12	F	4	180	6	8	700	35	NR	8.7	890	90	PR	12	1900	190	CR		12	2500	210	CR
6	12	M	5	230	16	6.2	600	39	NR	5	550	60	NR	7	700	70	PR	RLE	-	-	-	BMT
7	15	M	5	189	8	9.7	1400	111	PR	11	1560	130	CR	12	2900	150	CR		-	-	-	Rel
8	5	M	5	700	15	11	4123	126	CR	11	4300	155	CR	11	3000	200	CR		11.3	2500	209	CR
9	4	M	5	171	9	6.5	780	48	NR	7	800	70	PR	7.5	850	60	PR		-	-	-	BMT
10	9	F	5	280	5	5	713	30	NR	5	580	30	NR	7	600	40	NR		-	-	-	Died
11	11	F	4.5	324	12	5.2	365	14	NR	5.9	567	34	NR	6.9	678	23	NR		-	-	-	BMT
12	13	F	5.3	435	11	5.6	893	45	NR	8.2	845	67	PR	11.2	2890	123	CR		12.6	3452	198	CR
13	12	M	6.2	324	9	10	1364	56	PR	8.6	876	89	PR	9.7	987	109	PR		9.2	1234	91	PR
14	6	M	5	345	8	4.9	389	34	NR	6.8	534	31	NR	10.4	1004	96	PR	He	-	-	-	BMT
15	13	M	5.6	294	3	6.7	402	28	NR	8.7	987	72	PR	10.9	985	123	PR		10.1	1234	213	CR
16	13	M	4.7	478	16	8.4	1211	87	PR	12	1301	132	CR	11.9	1356	145	CR		-	-	-	Leuk
17	14	M	4.4	189	4	9.1	1390	101	PR	11.8	1453	113	CR	12.2	2134	132	CR	H	11.6	3298	145	CR
18	4	F	7.2	134	6	7.9	458	14	NR	6.3	398	19	NR	6.8	354	29	NR	-	-	-	-	Died
19	4	M	6.3	456	14	11.1	2678	134	CR	10.9	1987	137	CR	12.8	2123	213	CR	GH	12.3	2134	167	CR
20	10	F	3.4	413	12	5.7	453	27	NR	10.6	832	58	PR	11.4	1134	78	PR	RLE	-	-	-	BMT

*R* response; *SE* side effects; *GH* Gum hyperplasia; *SR* skin rash; *RB* raised bilirubin; *RLE* raised liver enzymes; *He* headache; *H* hirtutism; *Rel* relapse; *Leuk* leukemia

**Table 3:** Summary of response rate of CsA only group

SN	Age (yr)	Sex	Before CSA			At 3 months of CsA			Response	At 6 month of CSA			response	At 12 month of CSA			Response	SE
			Hb (g/dl)	ANC (cell/ul)	PLT (x109/L)	Hb (g/dl)	ANC (cell/ ul)	PLT (x109/L)		Hb (g/dl)	ANC (cell/ul)	PLT (x109/L)		Hb (g/dl)	ANC (cell/ul)	PLT (x109/L)		
1	6	M	4	300	4	5	660	42	NR	7	600	30	NR	-	-	-	died	RD
2	12	F	4.8	615	19	6	896	29	NR	6	500	25	NR	7	780	38	PR	H
3	11	M	4	220	6	6	110	9	NR	6	380	19	NR	5	400	20	NR	
4	10	M	6	330	19	8	590	28	NR	8	500	30	NR	10.9	800	70	PR	
5	8	M	2	200	5	4	280	8	NR	5	490	24	NR	7	500	30	NR	
6	17	M	4.5	187	9	5	656	27	NR	5	500	20	NR	6	500	38	NR	
7	15	M	4	737	28	6.8	1200	39	NR	6.8	1300	33	PR	12	2000	100	CR	H
8	11	M	4	170	8	5	259	19	NR	6.8	380	20	NR	6	460	33	NR	
9	9	M	5	700	22	6.3	769	28	NR	7	1000	35	PR	11	1900	120	CR	
10	6	F	5	200	7	6.7	380	19	NR	-	-	-	died	-	-	-	died	

*SE* side effects; *RD* renal dysfunction; *H* hirtutism

### Hematological response after 3 months of treatment

At the 3 months evaluation, two patients in the E-PAG + CsA group (10% ) fulfilled the hematological criteria for CR and no longer required transfusion of packed red blood cells (PRBCs) or platelets. Six more patients (30%) in this group achieved PR. All six were still PRBC transfusion-dependent but no longer required platelet transfusion The remaining twelve (60%) E-PAG + CsA patients did not meet any of the response criteria and were still transfusion-dependent. In contrast, none of the 10 patients in the CsA group fulfilled the criteria for hematological response and all (100%) continued to require regular PRBC and platelet transfusions Additionally, ANC, Hg and platelets (Table 4) were significantly higher in the E-PAG group than the CsA group (*p* = 0.04, *p* = 0.01, *p* = 0.009, respectively).

**Table 4:** CBC follow-up after 3, 6, and 12 months of treatment

Character	CsA (*n*= 10)	E-PAG+ CsA group (20)	*P*-value
After 3 months			
ANC	623 (274.7- 800.7)	800 (537.5- 1420)	**0.04**
Hg	6 (5 - 6.7)	7.7 (6.1- 9.05)	0.01
PLT	27.5 (16.5 – 38.2)	42.5 (30 - 94)	0.009
After 6 months			
(*n*=9 )		(*n*=20)	
ANC	500 (435 - 800)	860.5 (590- 1451)	**0.01**
Hg	6.8 (5.5 - 7)	8.65 (6.9 – 10.9)	**0.01**
PLT	25 (20 – 31.5)	84.5 (59- 121.5)	**0.004**
After 12 months			
(*n*=8)		(*n*=20)	
ANC	640 (470 - 1625)	1245 (775-2128.5 )	**0.04**
Hg	7 (6 – 10.9)	11 (7.75 – 11.98)	**0.047**
PLT	38 (30.7 – 92.5)	116.5 (74 - 150)	**0.01**

Data are expressed as the number of cases (%) or median and inter-quartile range. Note: *p*-Value is calculated to compare both cohorts using Mann-Whitney test.

### Hematological response after 6 months of treatment

At the 6-month assessment a CR was found in seven (35%) of the E-PAG + CsA patients and PR in another eight patients (40%), all were transfusion-independent but three still required transfusion of blood components.

The remaining five E-PAG+ CsA patients (25%) showed no response to the combination therapy. In the CsA group, only two patients (20%) met the PR criteria and seven showed no response to treatment (Table 3). One patient died of a severe infection in the fourth month of CsA. All of the remaining nine were still PRBC and platelet transfusion-dependent. The highest response rate in the E-PAG+ CsA group was associated with a significant increase in ANC, Hb and platelet counts (Table 4) compared to that in the CsA group (*p* = 0.01, 0.01 and 0.004, respectively).

### Hematological response after 12 months of treatment

At 12 months, the number of patients in the E-PAG+ CsA group who respond to treatment increased to 17. Ten (50%) patients fulfilled the CR criteria, and seven (35%) patients had PR. All of them had become independent of platelet transfusion but three patients of PR still required PRBCs transfusion.

Three patient (15%) in the E-PAG+ CsA group had not responded to the therapy and was still dependent on transfusion support and waiting for a bone marrow transplant (BMT) (Table 2). In the CsA group, another patient died of a massive intracranial hemorrhage (Table 3). Two of the eight patients who had previously attained PR after 6 months of treatment achieved CR (25%) and were no longer transfusion-dependent. Another two patients displayed PR (25%), with one still dependent on PRBC and platelet transfusions every 8 weeks and the other requiring only platelet transfusion. Lastly, four patients in this group did not fulfil any of the hematological response criteria and were still transfusion-dependent. Furthermore, ANC, Hb and platelet were significantly higher in the E-PAG+ CsA group than the CsA group (*p* = 0.04, *p* = 0.047, and *p* = 0.01, respectively) (Table 4).

### Overall response and survival rates

At 3 months, the OR rate in the E-PAG+ CsA group was 40% (10% CR and 30% PR) which differed significantly from 0% in the CsA group (*p* = 0.006). At 6 months, CRs in the E-PAG+ CsA had risen significantly to 35% (*p* = 0.0001), with an 75% OR rate. The OR rate in the CsA group had increased to 20% (Table 5).

**Table 5:** Response rat and overall survival rate in the studied groups

	E-PAG+ CsA group (*n*= 20)					CsA group (*n*=10)					*P* value
	CR N/%	PR N/%	NR N/%	OR N/%	Death (N/%)	CR N/%	PR N/%	NR N/%	ORR N/%	Death (N/%)	
At 3 months	2(10%)	6 (30%)	12(60%)	8(40%)	-	0	0	10(100%)	0 (0%)	-	*006
At 6 months	E-PAG group(*n*=20)					Historical group (10)					
	7(35%)	8(40%)	5(25%)	15(75%)	-	0	2(20%)	7(70%)	2(20%)	1(10%)	*0.0001
At 12 Months	E-PAG group(*n*=20)					Historical group (*n*=9)					
	10(50%)	7(35%)	3(15%)	17 (85%)	-	2(25%)	2(25%)	4(50%)	4(50%)	1(10/%)	*0.0001
Overall survival at 12 ms	20 (100%)					8 (80%)					

*CR* complete response, *PR* partial response, *OR* overall response, *NR* no response

*P-value is calculated to compare the CR rate in both cohorts using Z Score test

Lastly, at the 1-year evaluation, the OR was 85% (50% CR and 35% PR) in the E-PAG + CsA group compared to 50% (25% CR and 25% PR) in the CsA group (*p* = 0.001). The overall survival rate at 1 year was 100% in the E-PAG+ CsA group and 80% in the CsA group.

### Side effects and clonal evolution

Overall, both treatment arms had acceptable toxicities. None of the patients had to withdraw from the study due to adverse events. The most common adverse event in the E-PAG+ CsA group was indirect bilirubin elevation (*n* = 3, 15%). Two patients (10%) showed transient elevation of their liver enzyme levels and two patients experienced headaches. The abnormal levels of bilirubin and liver enzymes self-resolved or disappeared after transient E-PAG dose adjustment. Hirsutism, a known CsA side effect, occurred in two E-PAG+ CsA patient. Asymptomatic gum hypertrophy was noted in one patient but did not require a decrease in drug dosage (Table 2). In the CsA group, mild renal dysfunction was seen in one patient but this subsided after decreasing the CsA dose to 5mg/kg for two weeks. Hirsutism occurred in two patients (Table 3).

### Long-term outcomes (at 24 months) of the E-PAG+ CsA group

At the 2-year evaluation of the E-PAG+ CsA group, eight ( 40%) of the patients who had responded completely still fulfilled the CR criteria without the need for transfusion support, while one patient (5%) had clinical signs of clonal evolution and one had relapse (5%). One of the seven PR patients now met the CR criteria while four patients (20%) had undergone BMT since the previous follow-up. The other two PR patients, their parents refused the BMT. The two E-PAG patient (10%) who had not responded to therapy had died of severe infection before reaching the top of the BMT waiting list while one had undergone BMT. There was no 2-year follow-up of the CsA group due to the retrospective data collection for that group.

## Discussion

CsA has been used to treat AA patients lacking a donor, the financial means, or medical eligibility for HSCT since the 1980s. CsA is a potent immunosuppressant. It is inexpensive, easily available, and non-myelotoxic. CsA exerts its effects by suppressing early T cell activation, inhibiting lymphokine production [32, 33]. Several studies have demonstrated the effectiveness of CsA in the treatment of SAA [34, 35]. It has a 50% response rate in SAA refractory to treatment with ATG or anti-lymphocyte globulin (ALG) [36, 37].

In the present study, we evaluated CsA monotherapy in 10 children with SAA. The response rates were 20% and 50% after 6 and 12 months, respectively, in eight of the 10 patients (due to a mortality rate of 20%). These figures support those found in a previous retrospective evaluation of CsA monotherapy in 66 children with AA, which found a 47% response rate in SAA cases over a period ranging from 2 to 34 weeks [38]. Another study of 44 children evaluated combined CsA + corticosteroid treatment of AA. They reported a mortality rate of 44.9% (18 patients). 42.3% of the surviving patients showed a CR but most had non-SAA. After 18 months, the response rate was 34.6% [39].

Our results differ from those found in studies of CsA treatment for adults with SAA. Shetty et al. recorded a 56.2% response rate to CsA in 20 adult patients with SAA after 3 months39, while Ghazaly et al. found a 50% response in adults with SAA at 6 months [15].

E-PAG is a thrombopoietin receptor agonist that has proven effective in adults with AA. When combined with IST, E-PAG is well-tolerated, and produces improved response rates, recovery of blood cell counts, and restoration of trilineage haematopoiesis, even after drug discontinuation. E-PAG has recently been approved for use as a first-line treatment for adult patients with SAA in combination with standard IST28.

Studies to evaluate the efficacy of E-PAG in pediatric SAA are rare and most assess its use of E-PAG with standard IST of ATG + CsA (Table 6) [29, 40–42].

**Table 6:** Comparison of research evaluating eltrombopag + immunosuppressive therapy as a treatment for severe aplastic anemia

Follow-up Number ()	Present study E-PAG + CsA (10/10) pediatric	Jie et al.^41^ E-PAG + IST 14 pediatric	Fang et al.40 E-PAG + IST (18/57) pediatric	Lesmana et al.^30^ E-PAG + IST (25/25) pediatric	Scheinberg et al.^42^ E-PAG + CsA (54 adults)	Townsley et al.27 E-PAG + IST (19/92) pediatric
Three months						
ORR (%)	40%	35.7%	77%	-	40.7%	80%
CRR (%)	10%	7.1%	22.2%	-	-	30%
Six months						
ORR (%)	75%	78.5%	94.4%	77.7%	46.3%	87%
CRR (%)	35%	64.3%	50%	29%	5.4%	
Twelve months						
ORR (%)	85%			100%		
CRR (%)	50%			58%		

*CRR*, complete response rate; *CsA*, cyclosporine; *E-PAG*, eltrombopag; *IST*, immunosuppressive therapy; *ORR*, overall response rate

To the best of our knowledge, this is the first prospective study to evaluate the efficacy and safety of E-PAG in combination with CsA alone in children with SAA.

In the present study, the OR rate of E-PAG patients was 40% after 3 months of therapy, with two patient achieving CR and six, PR. At 6 months, the OR was 75% with a CR of 35% and most of these patients were independent of transfusion support. In contrast, the CsA group experienced 20% PR at 6 months. After a year of regular treatment, the rate of complete responses to the E-PAG+ CsA regimen had increased to ten patients (50%), with an OR rate of 85%. The survival rate at 12 months was 100% compared to 80% in the CsA cohort. Additionally, the E-PAG+ CsA group experienced a 90% survival rate at 24-month follow-up, although one of the survivors underwent a hematological relapse. Elevated transaminase levels or indirect elevation of bilirubin levels occurred in five of the patients treated with E-PAG+ CsA. Renal insufficiency occurred in one CsA patient. The elevations and renal insufficiency were corrected with dose adjustments. Hirsutism, a known side effect of CsA, was observed in both cohorts.

Lesmana et al. [29] conducted a retrospective comparison of children with SAA treated with IST and E-PAG and those treated with standard IST. The CR rate in the IST and E-PAG group was 29% at 6 months and 58% at 12 months, while the OR rate was 77.7% at six and 100% 12 months. However, they recorded a significantly higher rate of renal insufficiency and elevated transaminase in the E-PAG cohort. These results were somewhat similar to our own, which may suggest that E-PAG and CsA exert similar effects to E-PAG and IST.

Another recent study of the efficacy and safety of E-PAG as a first-line treatment of pediatric AA found CR and OR rates at 6 months of 64.3% (9/14 case) and 78.6% (11/14 cases), respectively, with a 100% survival rate at [24] months and no relapse or intolerable side effects. They found a significantly higher rate of CR rate in SAA children treated with E-PAG + IST than that seen in the historical cohort [41]. This data shows a notable convergence between the results of the different treatment regimens used in that study and this one.

Fang et al., [40 ] found IST + E-PAG to be more effective than IST alone in children with SAA. The CR and OR were significantly higher in their IST + E-PAG group than their IST group after 6 months (CR: 17.9% vs. 50%; *p* < 0.05, OR: 69.2% vs. 94.4%, *p* < 0.05). The present study found roughly equivalent, significantly higher CRs and ORs, in children treated CsA + E-PAG to the outcomes obtained with children treated with IST + E-PAG.

In a study of Scheinberg et al., [42] 54 treatment-naïve adults with SAA treated with E-PAG + CsA for 6 months, the goal OR rate was met by 46.3% of patients with 5.4% achieving CR. The OR rate at 3 months was 40.7%. Townsley et al. [27] conducted a prospective study of a cohort of 92 patients, 19 of whom were children, treated with IST + E-PAG. This study divided patients into three cohorts according to the day of E-PAG treatment initiation. The third cohort in whom E-PAG treatment was initiated on day one reported an OR rate of 80% and 87% after 3 and 6 months, respectively, while the CR was 30% at 3 and 6 months. They found the beneficial effects of E-PAG to be directly proportionate to the length of exposure to the drug. In addition to the hematologic response, bone marrow was found to be highly enriched with hematopoietic stem cells and multipotent progenitors after three and 6 months of therapy. Moreover, Hwang et al. [43], investigated E-PAG in AA patients on a non-trial all-comer basis over a 4.5-year period. They concluded that E-APG in AA patients was feasible, safe, and associated with very good responses. Instead, Groarke et al. evaluated whether the addition of EPAG to IST enhanced ORR in pediatric SAA. There was no significant difference in survival between the pediatric EPAG group and either the pediatric IST group. They concluded that, addition of eltrombopag to IST did not afford any clear therapeutic advantage to pediatric patients with SAA [44].

IST in the form of ATG and CsA is well-established as an alternative treatment for patients with SAA when an HLA-matched familial donor is not available [10, 12, 13, 45]. However, in healthcare systems with inadequate resources, few patients can afford such an expensive combination. E-PAG + CsA is an available, safe, easily monitored treatment option for pediatric SAA in developing nations where economic considerations are paramount. Combined cyclosporine + eltrombopag was found to be an effective and safe alternative treatment for pediatric SAA, particularly in countries with limited resources. This study was limited by its small sample size and the lack of similar studies in pediatric groups. A larger prospective study with longer follow-up is essential to evaluate response stability
